# Comparison of Signal Processing Methods for Reducing Motion Artifacts in High-Density Electromyography During Human Locomotion

**DOI:** 10.1109/OJEMB.2020.2999782

**Published:** 2020-06-03

**Authors:** Bryan R. Schlink, Andrew D. Nordin, Daniel P. Ferris

**Affiliations:** J. Crayton Pruitt Family Department of Biomedical EngineeringUniversity of Florida 3463 Gainesville FL 32608 USA

**Keywords:** Canonical correlation analysis, high-density electromyography, locomotion, motion artifact, principal component analysis

## Abstract

*Objective:* High-density electromyography (EMG) is useful for studying changes in myoelectric activity within a muscle during human movement, but it is prone to motion artifacts during locomotion. We compared canonical correlation analysis and principal component analysis methods for signal decomposition and component filtering with a traditional EMG high-pass filtering approach to quantify their relative performance at removing motion artifacts from high-density EMG of the gastrocnemius and tibialis anterior muscles during human walking and running. *Results:* Canonical correlation analysis filtering provided a greater reduction in signal content at frequency bands associated with motion artifacts than either traditional high-pass filtering or principal component analysis filtering. Canonical correlation analysis filtering also minimized signal reduction at frequency bands expected to consist of true myoelectric signal. *Conclusions:* Canonical correlation analysis filtering appears to outperform a standard high-pass filter and principal component analysis filter in cleaning high-density EMG collected during fast walking or running.

## I. Introduction

Bipolar surface electromyography (EMG) has long been the gold standard for recording electrical activity from muscles during locomotion [1]. Although a bipolar recording is the most widely-used configuration [2], its scope is limited to a single recording site on the target muscle. The signal recorded from this site is a summation of many muscle fibers and motor units active in that area [3]. There are spatial variations in EMG activity recorded from several sites on the same muscle, and small deviations in electrode placement can lead to different conclusions regarding muscle function [4]–[6].

**Fig. 1.**
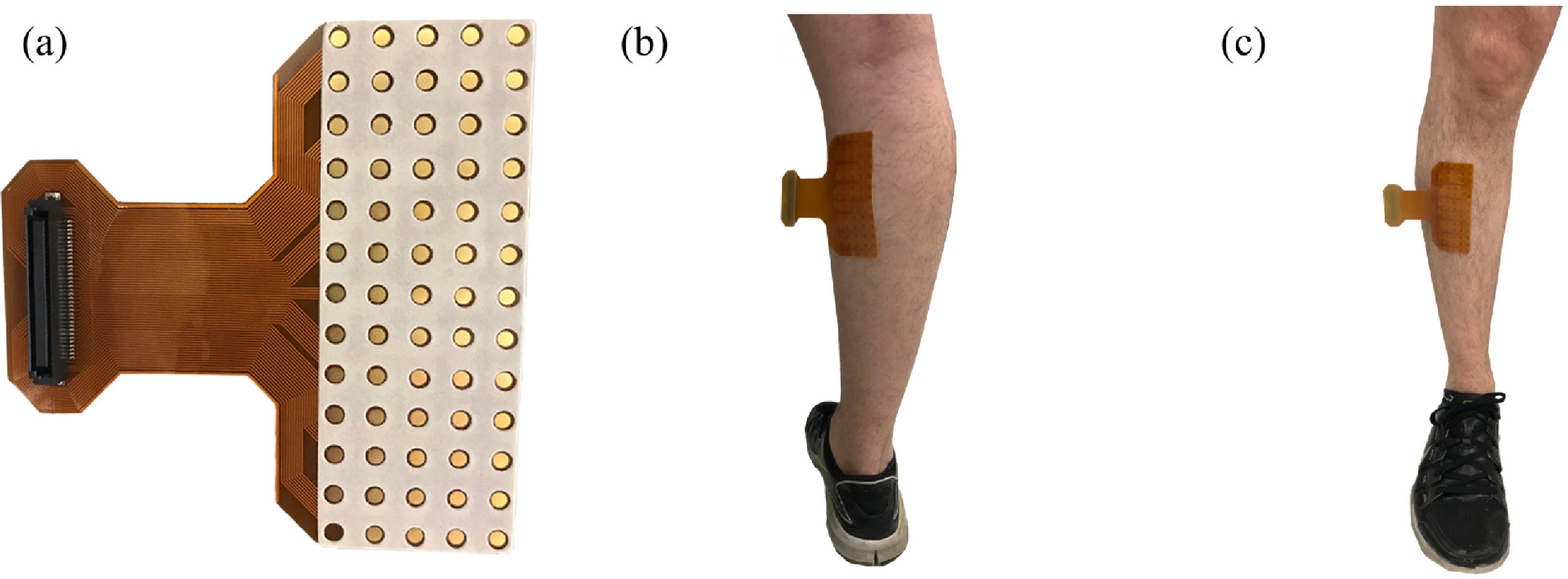
We placed 64-channel electrode arrays (a) on the right medial gastrocnemius (b) and tibialis anterior (c) muscles of each subject. Each array was secured to the leg using tape and athletic wrap (not pictured).

In contrast, high-density EMG is an emerging technology that uses an array of electrodes to measure both the spatial and temporal properties of a muscle [7]. High-density EMG provides greater coverage of the target muscle, and the small interelectrode spacing allows the user to record individual motor units across the entire muscle [8] (Fig. 1). It is typically used in a wide range of motor tasks to record spatial muscle activity [9] and detect motor unit activation patterns [10] under relatively stationary conditions where motion artifacts are limited.

Recently, studies have shown the potential of high-density EMG in more dynamic settings. Schmitz *et al.* measured high-density EMG from the vastus medialis during stationary cycling and found that the muscle fiber conduction velocity was relatively constant during fatiguing exercise [11]. Cronin *et al.* recorded high-density EMG activity at a self-selected walking speed and showed that lateral gastrocnemius activity varied spatially in healthy adults [12]. Hegyi *et al.* measured hamstring muscle activity with a linear array of electrodes at near-maximal running speeds and found large individual differences in spatial activity patterns [13].

As running speed increases, so too does the frequency of motion artifacts that contaminate the EMG signal [14]. This leads to potential overlap between motion artifacts and the low-frequency content of the true muscle activity. For example, the typical frequency range of tibialis anterior EMG activity during the swing phase of running is 60–270 Hz, but there is also a brief period post-heel-strike where the activity has a lower frequency range (10–90 Hz) [15]. Current EMG processing standards recommend a high-pass filter cutoff frequency of 10–20 Hz [16], [17]. However, these recommendations pertain to bipolar EMG recordings with low levels of motion artifact. Our previous work with an electrical leg phantom showed that standard high-pass filtering does not fully remove artifacts from high-density EMG recordings [18]. There are currently no established standards for filtering high-density EMG signals during running. Current high-density EMG hardware is often wired and bulky. This forces the subject to be tethered via cables, increasing the potential that motion artifacts will be present in the signal. Therefore, there is a need to develop appropriate data processing standards for high-density EMG running data.

High-density EMG provides an opportunity to leverage statistical processing techniques that are typically unavailable with bipolar EMG recordings. Principal component analysis is often used to extract only the important information from a large set of variables and express it in reduced dimensions [19]. It has been used on stationary high-density EMG recordings for accurate force estimation [20] and control of myoelectric prostheses [21]. Canonical correlation analysis uses blind source separation techniques to isolate contaminated components of a set of variables from clean components of the same set [22]. Al Harrach *et al.* showed that canonical correlation analysis can improve the signal-to-noise ratio of high-density EMG recordings during seated isometric tasks [23].

Principal component analysis and canonical correlation analysis have also been effective at removing motion artifacts from high-density electroencephalography (EEG) recordings during walking. Both EEG and EMG recordings are susceptible to motion artifacts from movement at the electrode-skin interface, cable sway, and movement of system components [16], [24]. Spectral overlap of the artifact sources and underlying biological signals can make noise removal challenging, although the relevant frequency range in EEG analyses is often lower than that of EMG analyses. The typical EEG signal-to-noise ratio is often low and contaminated with noise as well [25]. Despite these challenges, statistical decomposition methods have been able to selectively parse signal from noise in EEG recordings [26], [27]. Due to the relatively high signal-to-noise ratio of EMG recordings, these techniques may have value at removing motion artifacts from dynamic high-density EMG data.

The purpose of this study was to compare the results of different signal processing techniques on high-density EMG data during locomotion. We recorded data from two lower limb muscles in healthy individuals walking and running on a treadmill at a range of speeds. We processed their data using three different methods: (1) Standard high-pass filtering of the data with a 20 Hz cutoff frequency; (2) Monopolar and differential EMG channel cleaning using principal component analysis and component filtering; and (3) Monopolar and differential channel EMG cleaning using canonical correlation analysis and component filtering. Based on its effectiveness at removing motion artifacts from high-density EEG recordings, we hypothesized that canonical correlation analysis filtering would result in the greatest reduction in motion artifacts. To our knowledge, this is the first study to investigate the effectiveness of different data processing methods on high-density EMG recorded during walking and running.

## II. Results

The average number of channels that were rejected from the high-density EMG recordings of each muscle decreased when using component decomposition filtering methods to clean the channel data prior to high-pass filtering (Table I). Both principal component analysis and canonical correlation analysis decreased the number of rejected channels across all speeds when compared to standard high-pass filtering. Canonical correlation analysis significantly reduced the number of rejected channels at lower running speeds (2.0 and 3.0 m/s; p < = 0.017) in the medial gastrocnemius and higher running speeds (3.0–5.0 m/s; p < = 0.038) in the tibialis anterior. Principal component analysis significantly reduced the number of rejected channels at only 2.0 m/s in the medial gastrocnemius (p = 0.018) and 3.0 and 5.0 m/s in the tibialis anterior (p < = 0.04). There were no pairwise differences in the number of rejected channels at any gait speed in either muscle between data processed with principal component analysis and canonical correlation analysis (p > 0.05 for all).

**TABLE I:** Average Number of Rejected Differential EMG Channels From the Medial Gastrocnemius and Tibialis Anterior Muscles Using Each Processing Method (Mean ± Standard Deviation)

	**Medial Gastrocnemius**			**Tibialis Anterior**		
Speed (m/s)	High-Pass Filtering	Principal Component Analysis	Canonical Correlation Analysis	High-Pass Filtering	Principal Component Analysis	Canonical Correlation Analysis
5	4.9±2.9	3.9±2.6	4.6±2.5	**4.1**±**2.8^^†^**	**1.9**±2.1^*^	**2.3**±1.3^*^
4	4.9±3.5	3.3±2.9	3.8±3.2	**3.9**±**3.3^†^**	2.1±2.5	**2.0**±2.2^*^
3	**3.8**±**3.4^†^**	2.9±2.8	**2.3**±2.0^*^	**3.4**±**2.7^^†^**	**1.4**±1.9^*^	**2.1**±2.8^*^
2	**4.3**±**3.2^^†^**	**3.1**±2.7^*^	**2.7**±2.2^*^	2.8±2.9	1.4±2.1	1.4±2.8
1.6	4.0±3.3	2.9±1.7	2.5±1.9	2.9±2.7	1.8±2.2	1.8±2.5
1.2	4.2±3.1	2.9±2.1	3.0±2.3	2.1±1.6	1.5±1.8	1.1±2.1

*pairwise difference (p < 0.05) with high-pass filtering method; ^pairwise difference (p < 0.05) with principal component analysis; †pairwise difference (p < 0.05) with canonical correlation analysis.

**Fig. 2.**
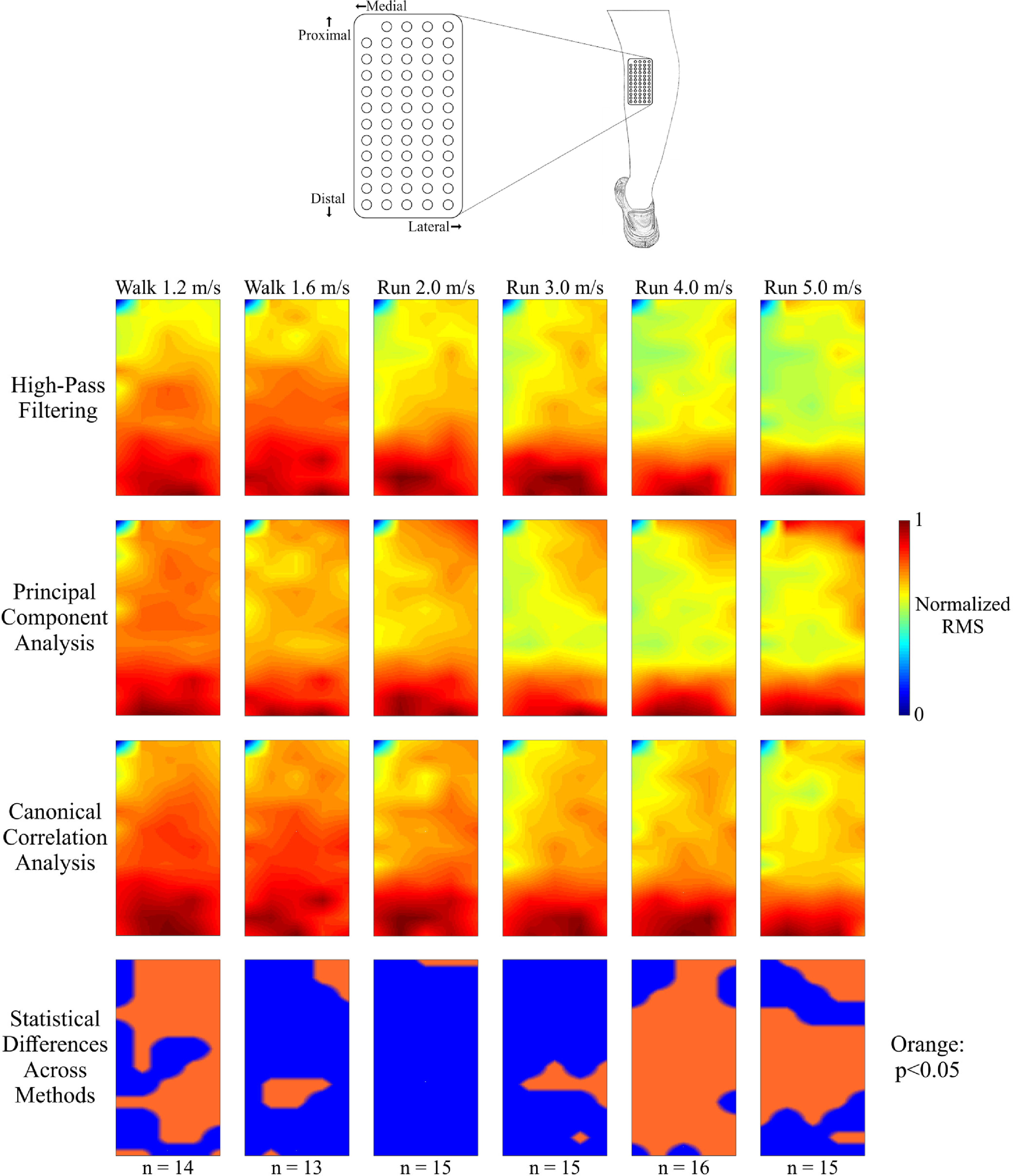
Spatial activation maps of the medial gastrocnemius muscle at each speed (columns) when processed using high-pass filtering (first row), principal component analysis (second row), and canonical correlation analysis (third row). The number of subjects whose data were included at each speed is denoted at the bottom (n). Data were normalized within each speed and processing method to the maximum root mean square (RMS) value of the differential EMG signals. Statistical differences in RMS value among the processing methods at each location are shown in the bottom row (orange: p < 0.05) after correcting for the false discovery rate. Every spatial map and statistical significance plot was interpolated by a factor of 8. All three processing methods show a focus of activation in the distal portion of the muscle. The central portion of the muscle had the greatest statistical differences across the three methods at the fastest speeds.

The greatest EMG activity in the medial gastrocnemius during stance was in the distal portion of the muscle, regardless of the speed and processing method applied to the data (Fig. 2). During walking, EMG activity was distributed across the measured surface of the muscle. At faster running speeds (3.0–5.0 m/s), electrical muscle activity was restricted to the distal portion of the muscle, where RMS values were nearly twice that of values in the middle of the array. Each processing method showed similar RMS spatial activity in each gait speed condition (Fig. 2, RMS plots). However, there were large numbers of electrode locations with significant differences in RMS amplitude at faster running speeds (Fig. 2, bottom: statistical difference plots). Among processing methods, normalized RMS amplitude differed at 50 of 59 electrode locations (85%) during 4.0 m/s running and at 39 locations (66%) during 5.0 m/s running (p < 0.05).

**Fig. 3.**
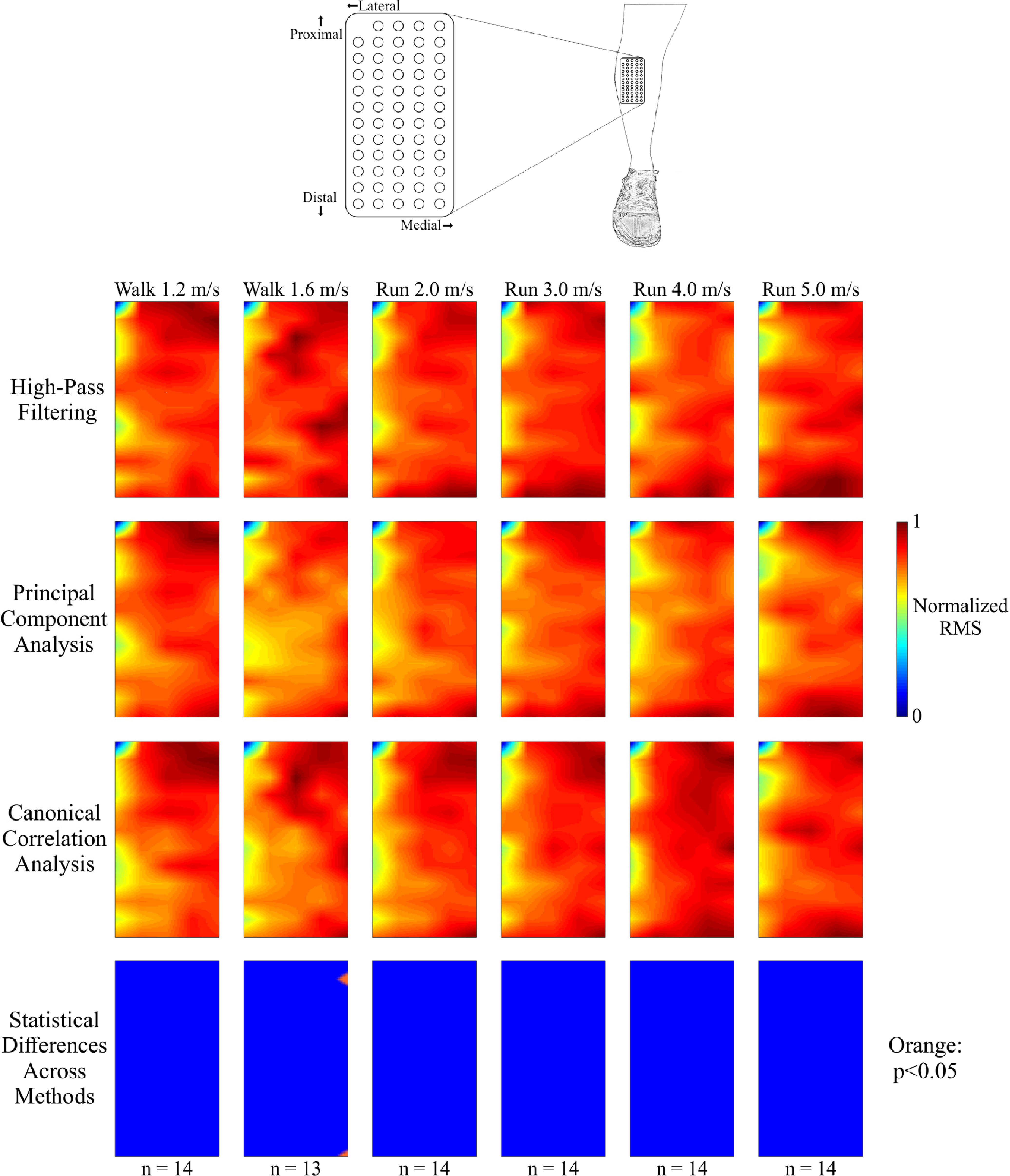
Spatial activation maps of the tibialis anterior at each speed (columns) when processed using high-pass filtering (first row), principal component analysis (second row), and canonical correlation analysis (third row). The number of subjects whose data were included at each speed is denoted at the bottom (n). Data were normalized within each speed and processing method to the maximum root mean square (RMS) value of the differential EMG signals. Statistical differences in RMS value among the processing methods at each location are shown in the bottom row (orange: p < 0.05) after correcting for the false discovery rate. Every spatial map and statistical significance plot was interpolated by a factor of 8. The spatial EMG activation was evenly distributed across the entire recorded muscle, regardless of processing method. An increase in EMG activity occurred at the distal portion of the muscle at faster running speeds. There were very few statistical differences in the RMS amplitude among the processing methods across all gait speeds.

Compared to the medial gastrocnemius muscle, the spatial distribution of the tibialis anterior EMG activity during swing was more evenly distributed across the measured surface of the muscle (Fig. 3). At slower gait speeds, the greatest EMG activity was in the proximal/medial region of the muscle. The EMG activity in the distal portion of the muscle increased with gait speed. Signal processing method had very little effect on the spatial RMS amplitudes for all walking and running speeds. The maximum number of locations with a significant difference in RMS amplitude was 1 (2%), during the 1.6 m/s walking condition.

**Fig. 4.**
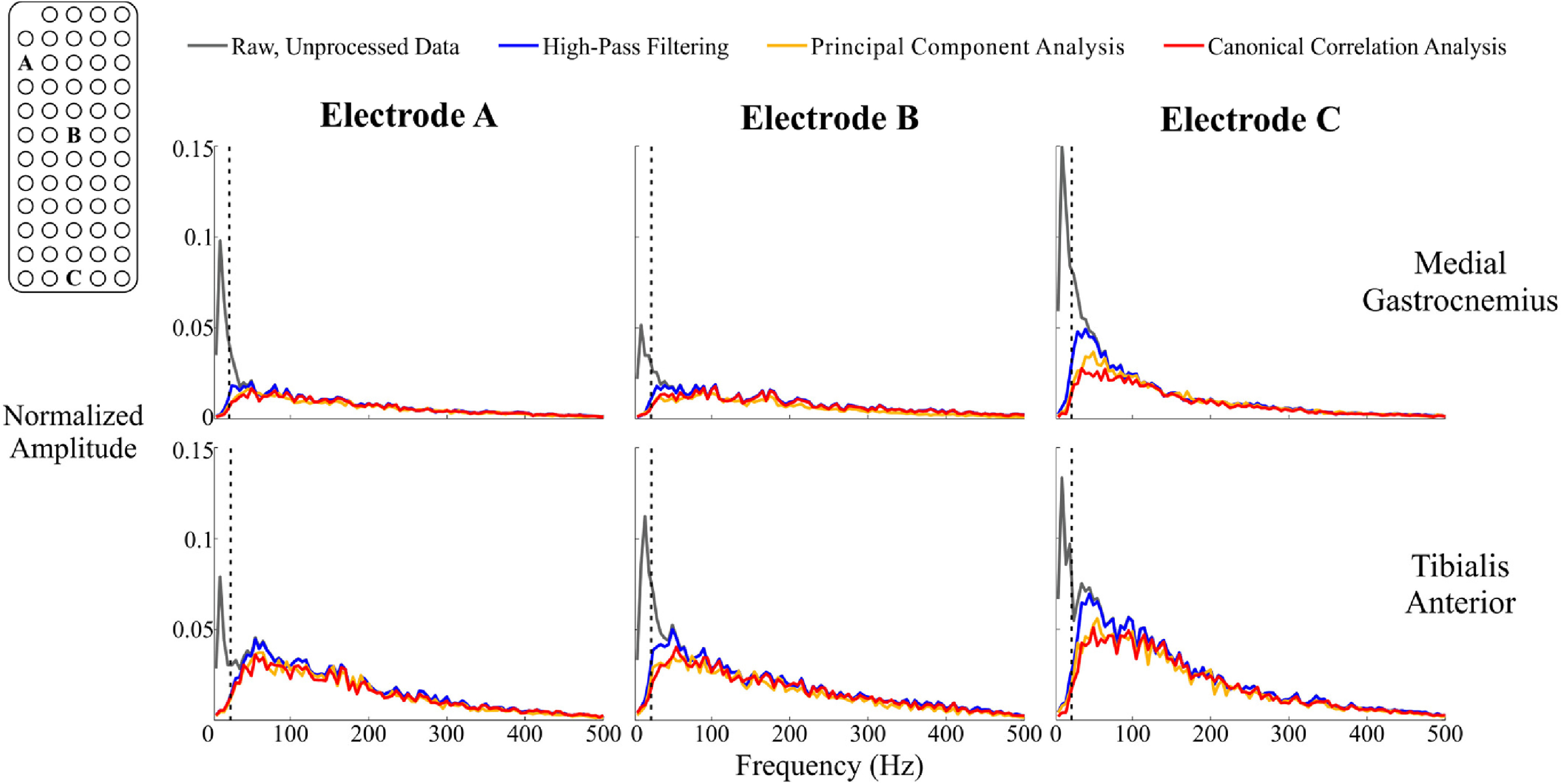
Exemplar frequency spectra from the medial gastrocnemius (top) and tibialis anterior (bottom) at three different electrode locations during running at 5.0 m/s. Data were normalized to the maximum amplitude value across all 59 differential electrode locations. The black vertical dashed line denotes the standard high-pass filter cutoff frequency (20 Hz). The distal portion of each muscle (Electrode C) had greater differences in spectral amplitude among the three different processing methods compared to the middle (Electrode B) and proximal (Electrode A) locations.

The frequency content of each differential channel varied based on the processing method that was applied (Fig. 4). There were pronounced spectral amplitude differences among processing methods in channels at the distal portion of each muscle (Fig. 4, Electrode C). Differential channels at the proximal and central portions of the electrode array had limited differences across all frequencies (Fig. 4, Electrodes A and B).

**Fig. 5.**
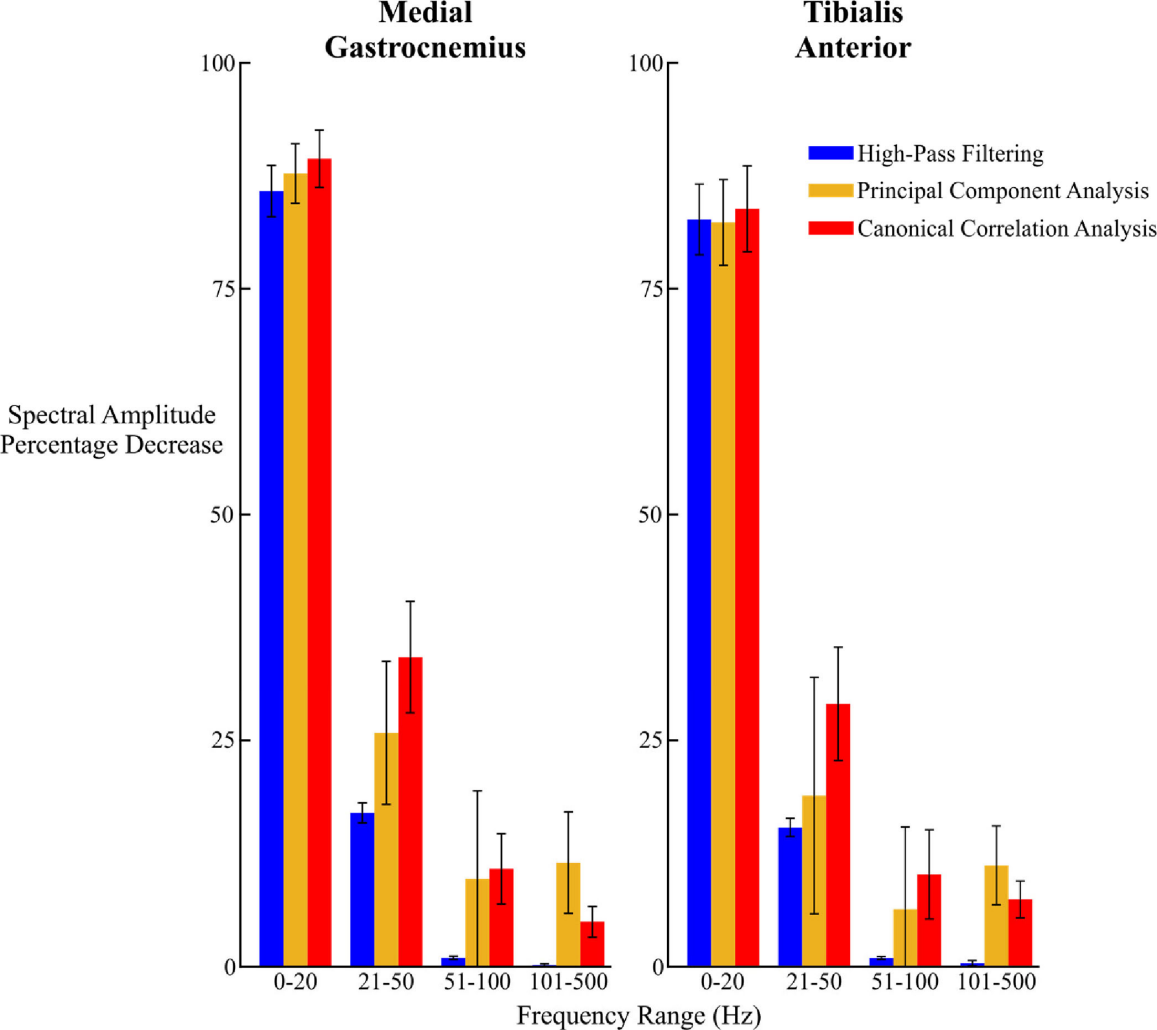
Average decrease in spectral amplitude between each processing method and the raw differential EMG channels in the medial gastrocnemius (left) and tibialis anterior (right) during running at 5.0 m/s. Error bars represent standard error. Canonical correlation analysis reduced the greatest amount of signal amplitude in frequency bands associated with motion artifacts (0–20 Hz; 21–50 Hz).

Compared to raw, unprocessed data, each processing method reduced the average overall low frequency spectral amplitude, which is predominantly associated with pure motion artifacts, during running at 5.0 m/s (Fig. 5, 0–20 Hz). In the intermediate frequency bands where both EMG signal and motion artifacts can reside, canonical correlation analysis produced the greatest average spectral amplitude reduction (Fig. 5, 21–50 Hz and 51–100 Hz). At higher frequencies, principal component analysis reduced the average spectral amplitude the most in each muscle (Fig. 5, 101–500 Hz). Principal component analysis also had the greatest variability in average spectral amplitude change for both muscles in all frequency ranges above 20 Hz. When the data were only high-pass filtered, the average signal amplitude decrease was less than 5% in all frequencies above 50 Hz. The spectral amplitude decrease for each method at all other speeds are shown in the Supplementary Material (Figs. 7–11).

## III. Discussion

Both principal component analysis and canonical correlation analysis component cleaning methods reduced motion artifact content from dynamic high-density EMG recordings more effectively than traditional high-pass filtering of channel data. Canonical correlation analysis reduced the average spectral amplitude more than the other two methods in frequency ranges typically associated with motion artifacts (Fig. 5). Principal component analysis had a greater reduction in signal amplitude in these frequency ranges, but it also removed more content in the higher frequency ranges that typically consist of mostly biological signal [28]. Although principal component analysis and canonical correlation analysis reduced the number of rejected channels in each muscle across all walking and running speeds, we observed very similar numbers of rejected channels across all speeds and processing methods. The type of processing method had very little effect in spatial EMG activity in the tibialis anterior at all speeds. This is likely due to the smaller muscle mass and reduced fat and cutaneous tissue on the anterior compared to the posterior portion of the lower limb.

Contrasting spatial EMG activity patterns were observed in the two muscles we measured. In the medial gastrocnemius, we found the highest electrical muscle activity amplitude in the distal portion of the muscle during stance phase at all walking and running speeds. Cronin *et al.* found a similar pattern in the lateral gastrocnemius during the stance phase of walking [12]. This localization of EMG activity may be explained by the regional grouping of muscle fibers and motor units found within this muscle [29], [30]. The EMG activity in the tibialis anterior muscle was more uniform across the high-density EMG array at all speeds, although we observed a restricted area of increased activity in the proximal region of the muscle during walking and slower running speeds. At faster running speeds (3.0–5.0 m/s), we observed regions of increased EMG activity in both the proximal and distal portions of the muscle. The differences in spatial EMG activation between these two muscles may be due to differences in muscle fiber types [31] or muscle architecture [32].

Although the spatial activation plots for each muscle appeared relatively similar after applying contrasting processing methods (Figs. 2 and 3), the frequency content of these signals were substantially affected by how the data were processed. Canonical correlation analysis reduced greater proportions of the signal amplitude compared to traditional high-pass filtering in both muscles, particularly in frequencies that are easily corrupted by motion artifacts (Fig. 5, 0-20 Hz range). The typical frequency spectrum of medial gastrocnemius EMG during locomotion ranges between 20-220 Hz, with the greatest activation intensity between 70-120 Hz [33]. Although principal component analysis also reduced the signal amplitude in lower frequency ranges (0–20 Hz, 21–50 Hz) compared to traditional high-pass filtering, it also had the greatest signal amplitude decrease at higher frequencies (101–500 Hz), which could contribute to a loss of myoelectric activity from the high-density EMG recording. The variability shown by principal component analysis in the reduction of spectral amplitude, especially in the 51–100 Hz frequency range is also undesirable. Conversely, canonical correlation analysis was less variable and reduced a smaller portion of the signal content in these ranges. Although motion artifacts rarely affect the upper frequency ranges of the EMG signal, broadband noise from the EMG amplifier can still contaminate the signal at higher frequencies [34]. It is possible that the low levels of signal reduction in the upper frequency ranges by canonical correlation analysis reduced this broadband noise.

Principal component analysis and canonical correlation analysis are analogous, but contrasting, methods that have each been used for isolating and removing noise sources from multivariate time series channel data [27], [35]– [37]. Applied to high-density EMG data, the relatively high signal to noise ratio of electrical muscle activity recordings allow much of the desired signal to be effectively separated from noise contamination using principal component analysis. However, because motion artifacts are coupled with electrical muscle activity during locomotion and can represent a large proportion of the signal variance, particularly at fast gait speeds, noise source separation using principal component analysis can be incomplete. In contrast, canonical correlation analysis relies on multiple correlations within a dataset to extract signal components based on their level of association. Derived based on autocorrelation here, canonical components also carry relationships with signal frequency content [36], [37]. Canonical components with very high autocorrelation can capture low frequency signal content attributed to motion artifact contamination, while components with low autocorrelation capture and separate muscle activity and electrical artifacts [27]. Source separation using canonical correlation analysis can therefore provide advantages for high-density EMG signal cleaning.

The physical structure of a high-density EMG system likely contributes to the amount and type of motion artifacts during dynamic conditions. Most high-density EMG systems are typically designed for use in stationary environments. Each electrode array required an attached small adapter unit that was connected to the main EMG amplifier via a 3-meter ribbon cable. Although we taped and wrapped the adapter units and corresponding cabling to the subjects’ legs in an attempt to minimize movement artifacts induced by equipment and cable motions, cable sway and electrode mass are known contributors to motion artifacts in high-density biopotential recording systems [24]. As a result, movement at the electrode-skin interface, an additional source of motion artifacts [16], could be greater than what is typically seen in recordings with traditional mobile bipolar EMG systems. As high-density EMG technology evolves, the influence of motion artifacts on EMG signal quality may reduce. Wireless data transmission will eliminate the effects of cable sway, and continued device miniaturization will help minimize mechanical artifacts between the electrode and skin.

There were limitations with this study. To simplify our analyses, we focused on the primary phases of the gait cycle in which each muscle is active (stance: medial gastrocnemius, swing: tibialis anterior). Because muscle activity amplitude and timing vary with gait speed [38], it is possible that spatiotemporal muscle activation patterns differ within and between additional sub-phases of the gait cycle, and among other muscles. We limited our high-density EMG processing comparison to two muscles in the leg with inertial and tissue properties that could vibrate at different frequencies compared to others during locomotion [39]. It is, therefore, possible that other major leg muscles (quadriceps, hamstrings, etc.) could experience different levels of motion artifacts when recorded with high-density EMG. We also acknowledge that we chose to normalize our average RMS values within each processing method and speed, which precluded studying the comparative effects of gait speed on EMG amplitude within a given method. Our intent, however, was to investigate how processing methods influence the spatial distribution and frequency content of high-density EMG measurements at a range of distinct gait speeds.

## IV. Conclusion

Our results demonstrated that high-density EMG canonical correlation analysis channel decomposition and component cleaning is likely the preferred method for processing high-density EMG during fast walking or running. It provided the greatest reduction in signal content within frequency bands expected to include motion artifacts while minimizing signal reduction within frequency bands expected to contain true myoelectric signal. Electrical phantoms using ground-truth EMG sources could help determine if this signal reduction correctly targeted pure motion artifacts. Future studies that examine a wider range of muscles and different high-density EMG systems would also help validate our conclusions.

## V. Materials and Methods

### A. Subjects

16 healthy volunteers (10 Males, 6 Females; mean age 23 ± 5 years) with no history of major lower limb injuries or neurological conditions completed this study. All subjects provided written informed consent before participating, and all procedures were in accordance with the Declaration of Helsinki and approved by the University of Florida Institutional Review Board.

### B. Experimental Protocol

Subjects walked at two different speeds (1.2 and 1.6 m/s) and ran at four different speeds (2.0, 3.0, 4.0, and 5.0 m/s) on an instrumented treadmill. We randomized the order of the speeds for each subject and recorded data for twenty strides per leg. For safety purposes, all subjects wore a lightweight upper-body harness that did not impede or alter their running mechanics.

We attached high-density EMG arrays (OT Bioelettronica, Turin, Italy) to the medial gastrocnemius and tibialis anterior muscles of each subject's right leg (Fig. 1). The electrodes of each array had an interelectrode spacing of 8 mm and were arranged into 13 rows and 5 columns, with one corner empty for a total of 64 electrodes. We located each muscle's boundaries using ultrasonography (ArtUs EXT-1H, Telemed, Vilnius, Lithuania) to ensure proper placement of the arrays. We shaved and cleaned the skin over each muscle using an abrasive paste and alcohol swabs. We applied a conductive paste to every electrode on each array without bridging neighboring electrodes. To standardize our EMG array placement, we aligned the center of the electrode array on the midline/belly of each muscle according to guidelines proposed by Hermens *et al.* [2]. We then placed flexible tape and athletic wrap over each electrode array to ensure good contact with the leg and transmission of signals throughout the experiment.

We sampled the high-density EMG monopolar data at 2048 Hz with a bandpass filter of 10-500 Hz. We excluded trials that were corrupted by equipment malfunctions. We recorded ground reaction forces from an instrumented split-belt treadmill (1000 Hz; Bertec, Columbus, OH). All subjects ran on only one belt of the treadmill across all speeds. We low- pass filtered the ground reaction force data at 40 Hz (4^th^ order Butterworth filter, zero lag) to extract gait event timings.

### C. Signal Processing Methods

**Fig. 6.**
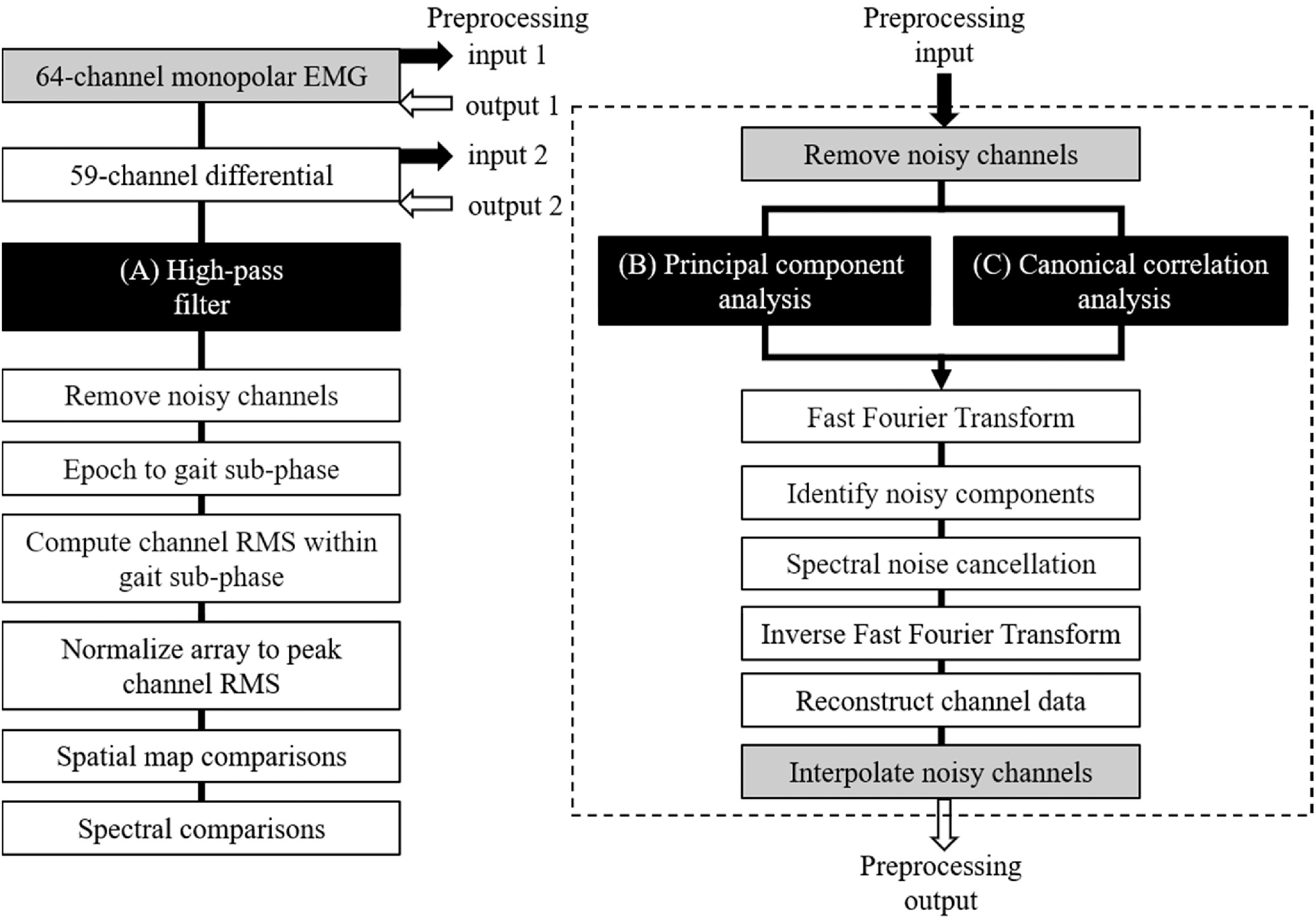
High-density EMG data signal processing pipeline. After down-sampling the raw 64-channel monopolar EMG recordings to 1000 Hz sample rate, channel data were processed using three different methods: (A) High-pass filtered (20 Hz), or preprocessed using statistical decomposition via (B) Principal component analysis, or (C) Canonical correlation analysis. When only applying the high-pass filter method (Left column), a 59-channel differential was computed among longitudinally neighboring channels in the array, followed by high-pass filtering the data, removing noisy channels based on statistical criteria, and extracting sub-phases of the gait cycle based on vertical ground reaction force events. The root mean square (RMS) of the EMG activity at each electrode was calculated and normalized to the peak channel RMS within the specified gait sub-phase to create mean electrical muscle activity spatial maps. When applying statistical signal decomposition preprocessing methods (Right column), the original 64-channel monopolar EMG signals (input 1) were screened for unusually noisy channels (statistical criteria), followed by (B) Principal component analysis or (C) Canonical correlation analysis. Using the component time series waveforms, Fast Fourier Transform (FFT) was applied and noisy components were identified based on statistical comparisons among components. Components with unusual spectral characteristics were subjected to spectral noise cancellation and reconstructed using the inverse FFT. Using both unaltered and cleaned components, the channel data were reconstructed and unusually noisy channels that were previously omitted were reconstructed using the neighboring channels (output 1). After computing the 59-channel differential, the same processing steps that were applied to the monopolar data were repeated on the differential data (input 2), without removing noisy channels or subsequent channel interpolation (Right column, grey boxes). From here (output 2), high-pass filtering was applied to the component-cleaned and reconstructed differential channel data followed by the remaining processing steps (Left column). The spatial maps from all three methods were compared using a one-way analysis of variance of the RMS values at each electrode location. The frequency spectra from each processing method were compared to the raw, unprocessed data at different locations along the electrode array. Spectral amplitude decreases from the raw, unprocessed data were also compared within specific frequency bands for each muscle and speed.

We applied three separate signal cleaning procedures to our high-density EMG channel data collected from medial gastrocnemius and tibialis anterior muscles using steps outlined in Fig. 6. In each case, we recorded monopolar EMG signals from the 64-channel array shown in Fig. 1. Prior to processing with each method, we down-sampled these monopolar signals to 1000 Hz to match the sampling frequency of our force plate data and to extract individual strides from the data. Fig. 6 shows common EMG processing steps used in each method. A detailed description of each method can be found in the Supplementary Materials.

### D. Methods Comparison

For each processing method, we calculated the number of rejected channels after high-pass filtering the data (Fig. 6, Left) using objective statistical criteria among channels, which have been adopted in high-density mobile EEG studies [40], [41]. This provides an unbiased method of channel rejection, which is often subjectively performed via visual inspection of the EMG signals [29], [38]. Further, because our goal was to compare the influence of processing methods on high-density EMG spatial activity, we normalized the activation amplitudes for each array to the peak of each respective gait speed condition, rather than scaling the array to a common measure among gait speed conditions. We investigated the effects of conventional and alternative processing methods on the spectral content of channel recordings in each gait speed condition to reveal frequency-specific amplitude changes at contrasting spatial locations on the high-density EMG array. To do so, we calculated the Fast Fourier Transform (FFT) of the signals at three different locations on each electrode array and evaluated the percent change in amplitude relative to the raw, unfiltered channel data. Because we present the aggregated normalized spectral amplitude among subjects and channels from the entire high-density EMG array, we excluded outlier channels (>10 scaled median absolute deviations among other channels within each subject) when calculating the mean EMG spectra (frequency vs. amplitude).

To statistically test differences among processing methods, we performed a one-way repeated measures ANOVA (α = 0.05) on the average number of rejected channels at each speed and the root mean square (RMS) values at each differential channel location. The latter allowed us to produce spatial statistical significance plots (p < 0.05) to reveal the influence of processing methods on high-density EMG spatial activity. To account for possible Type I error, we adjusted our results using a false discovery rate correction [42]. Ultimately, we were able to evaluate differences in spatial muscle activity amplitude and spectral content among processing methods.

## Supplementary Materials

Supplementary Materials
