# Supplementary material for: Comparison of Signal Processing Methods for Reducing Motion Artifacts in High-Density Electromyography During Human Locomotion
Source: IEEE Open J Eng Med Biol. 2020 Jun 3;1:156–65. doi: 10.1109/OJEMB.2020.2999782 (PMC8974705; doi:10.1109/OJEMB.2020.2999782)
Supplement: Supplementary Materials [file supp1-2999782.pdf]

Supplementary Materials

Comparison of Signal Processing Methods for Reducing Motion Artifacts in High-Density Electromyography during Human Locomotion

Bryan R. Schlink, *Member, IEEE*, Andrew D. Nordin, *Member, IEEE*, and Daniel P. Ferris, *Senior Member, IEEE*

We compared three different signal processing methods in this paper, further described in the Methods section below. We have also provided the average spectral amplitude decrease data for the 1.2, 1.6, 2.0, 3.0, and 4.0 m/s conditions.

Methods

1) High-Pass Filtering

For each muscle, we converted the 64 monopolar EMG signals into 59 differential signals along the longitudinal axis of the electrode array. We then filtered each signal using a 4th order high-pass Butterworth filter with a cutoff frequency of 20 Hz and zero lag. This is a standard cutoff frequency when high-pass filtering traditional bipolar EMG data [16] and has previously been applied to high-density EMG data recorded during locomotion [12], [13].

To avoid biasing our results from each preprocessing method, we adopted objective statistical criteria to remove EMG channels with poor signal quality (e.g. high baseline noise, poor skin contact, etc.) (Fig. 6). Our channel exclusion criteria were based on common high-density EEG channel rejection methods that identify signals with unusual statistical characteristics [40], [41]. Here, high-density EMG signals with large voltage fluctuations compared to the surrounding channels in the array were removed from further analysis. Based on exploratory pilot testing of the dataset, EMG channels with standard deviations that exceeded 4 scaled median absolute deviations from the median among channels were excluded.

We extracted epochs from the EMG channel data based on gait events to analyze phase-dependent activation patterns in our target muscles (medial gastrocnemius: stance, tibialis anterior: swing). For each subject, muscle, EMG channel, and gait speed condition, we separately calculated the root mean square (RMS) value during each gait sub-phase (stance and swing) and averaged these values among strides (Fig. 6). This provided an average muscle activation spatial map within the gait sub-phase for each subject, muscle, and gait speed. Based on the peak RMS value within the spatial map, we normalized the EMG array (0 to 1 scale) before averaging among subjects. After computing mean muscle activation spatial maps among subjects for each gait sub-phase and speed condition, we normalized the spatial map amplitude to the peak RMS across the array (0 to 1 scale). We interpolated these spatial maps by a factor of 8 to present a smoother representation of the EMG activity across the entire muscle (interpolated values were not used during statistical analysis).

2) Principal Component Analysis &
3) Canonical Correlation Analysis

Principal component analysis and canonical correlation analysis have each been used to isolate and remove noise sources from biopotential recordings [27], [35]–[37]. Principal component analysis derives components based on variance and canonical correlation analysis derives components based on correlations within a dataset. Here, we used these methods to preprocess the EMG data before using the standard high-pass filtering pipeline.

For each method, we first decomposed the 64-channel raw monopolar data into components (Fig. 6, Right). Prior to decomposing the high-density EMG channel data, however, we omitted monopolar channels with very large voltage fluctuations relative to the surrounding channels in the array (input 1, Fig. 6). Specifically, we excluded monopolar EMG channels with standard deviations that exceeded 20 scaled median absolute deviations from the median among channels. We selected these criteria based on exploratory pilot testing of the dataset to exclude unrecoverable channel data from our component decompositions. We subsequently interpolated monopolar channels that were omitted from the monopolar channel decomposition by averaging all surrounding monopolar channels on the electrode array.

Applying principal component analysis to our input matrix of EMG channels by time samples, we extracted principal component loading vectors and scores. Because loading vectors are time series representations of signal components, we used these variables to distinguish signal and noise components. In contrast, our canonical correlation analysis input consisted of the same input matrix used in principal component analysis (EMG channels by time samples), and the same matrix lagged by 1-frame. Canonical components were derived based on autocorrelation, to produce canonical component loading vectors and weights. Due to the relationship between autocorrelation and signal frequency content [36], [37], canonical components with very high autocorrelation captured low frequency signal content that can be attributed to motion artifact contamination [27].

In each high-density EMG decomposition approach, we evaluated the statistical characteristics of the components by decomposing the component time series into the frequency domain using Fast Fourier Transform, as outlined in Nordin et

al. [27] (Fig. 6, Right). Using the amplitude portion of the component spectra, we calculated skewness, kurtosis, and standard deviation among frequencies (0-500 Hz). Components with unusual spectral statistics (>2 scaled median absolute deviations among components) were further evaluated for noise contamination. In contrast to statistical channel rejection steps, component noise contamination was evaluated using statistical spectral features due to the frequency dependence of motion artifacts in high-density mobile EEG data [27]. Using this approach, we performed spectral noise cancellation (frequencies >10 times or <2 times the median spectral amplitude in each component) [27], [43]. After performing the inverse Fast Fourier Transform for each component, we reconstructed the high-density EMG channel data from the cleaned components (Fig. 6, Right).

We converted our cleaned monopolar data into 59-differential channels along the longitudinal axis of the array. We then took these differential channel data (Fig. 6, input 2) and used the same preprocessing steps that we had used on the monopolar channels. However, we did not find that channel rejection or interpolation were necessary for the differential channel decomposition. After the signal decomposition, cleaning, and subsequent reconstruction of the differential channels were complete (Fig. 6, output 2), the data went through the same process of high-pass filtering, noisy channel removal, and muscle activity spatial map preparation that were outlined for the high-pass filtering method.

RESULTS

Figs. 7-11 show the average spectral amplitude decrease of each method compared to the raw, unprocessed data during running at 4.0 m/s (Fig. 7), 3.0 m/s (Fig. 8), 2.0 m/s (Fig. 9) and walking at 1.6 m/s (Fig. 10) and 1.2 m/s (Fig. 11).

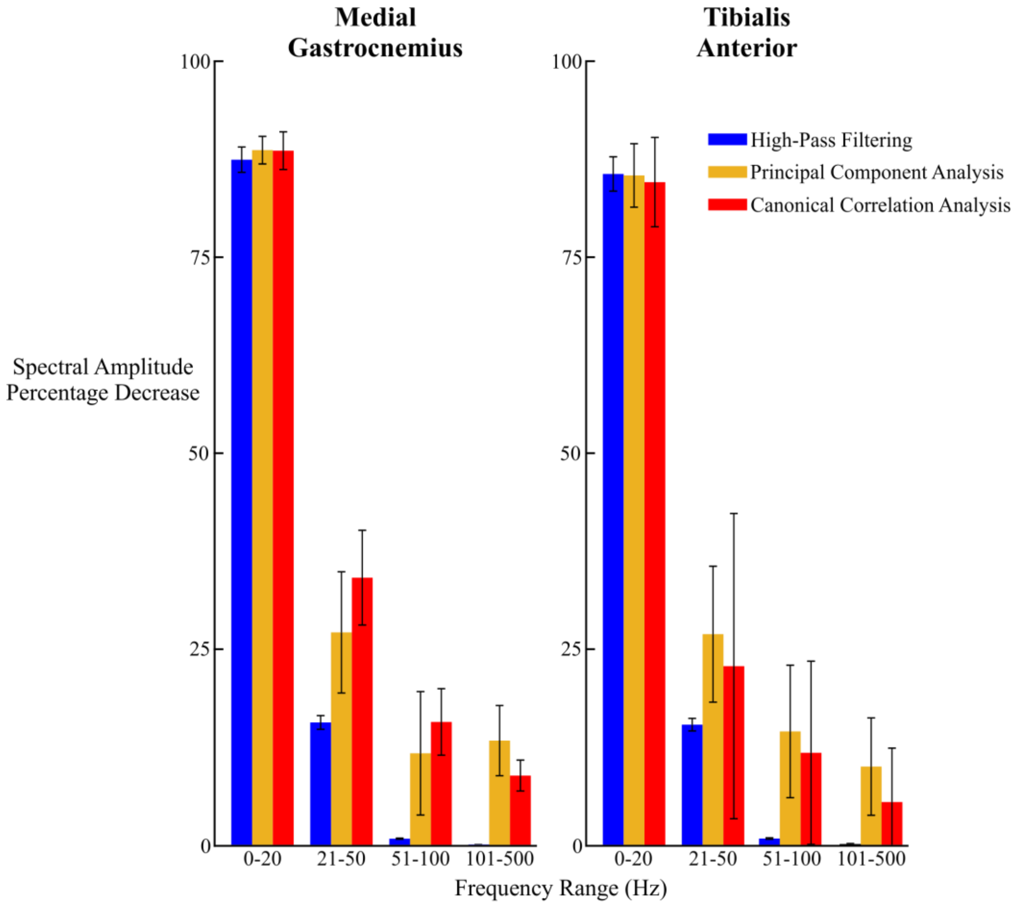

Figure 7. Average decrease in spectral amplitude between each processing method and the raw differential EMG channels in the medial gastrocnemius (left) and tibialis anterior (right) during running at 4.0 m/s. Error bars represent standard error. Canonical correlation analysis reduced the greatest amount of signal amplitude in frequency bands associated with motion artifacts (0-20 Hz; 21-50 Hz).

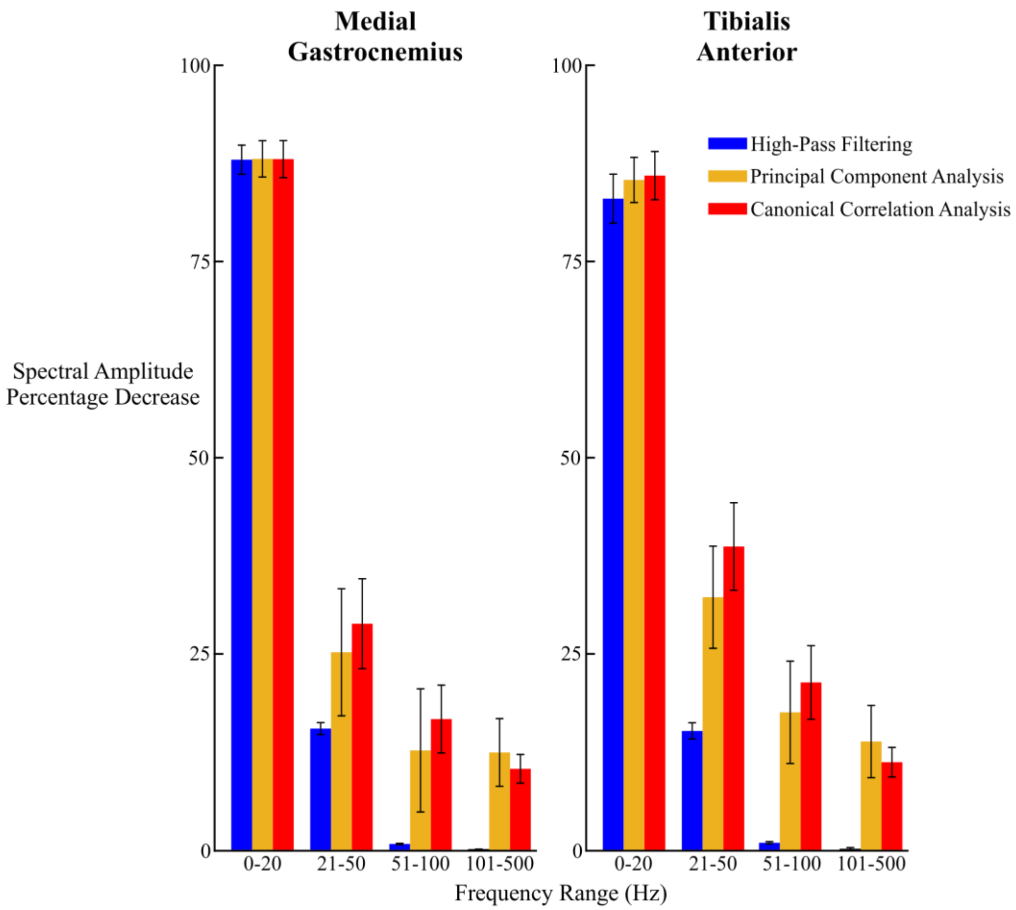

Figure 8. Average decrease in spectral amplitude between each processing method and the raw differential EMG channels in the medial gastrocnemius (left) and tibialis anterior (right) during running at 3.0 m/s. Error bars represent standard error. Canonical correlation analysis reduced the greatest amount of signal amplitude in frequency bands associated with motion artifacts (0-20 Hz; 21-50 Hz).

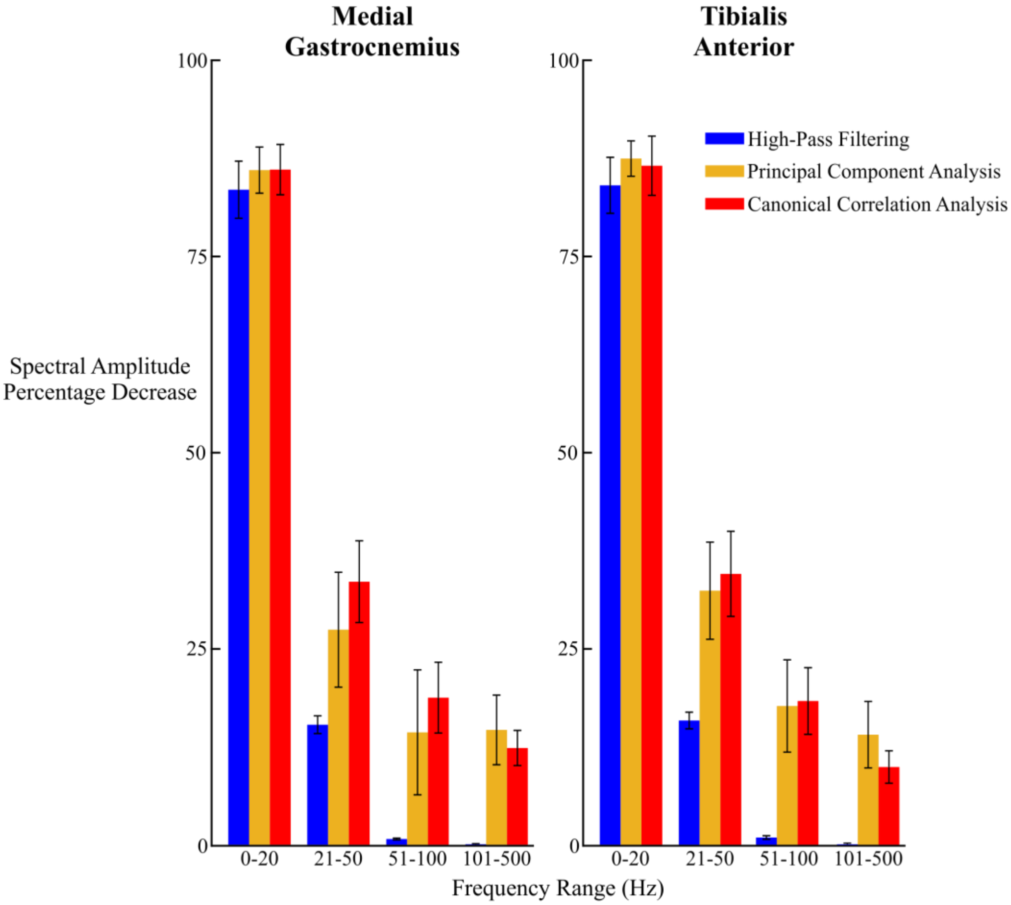

Figure 9. Average decrease in spectral amplitude between each processing method and the raw differential EMG channels in the medial gastrocnemius (left) and tibialis anterior (right) during running at 2.0 m/s. Error bars represent standard error. Canonical correlation analysis reduced the greatest amount of signal amplitude in frequency bands associated with motion artifacts (0-20 Hz; 21-50 Hz).

IEEE Open Journal of Engineering in Medicine and Biology

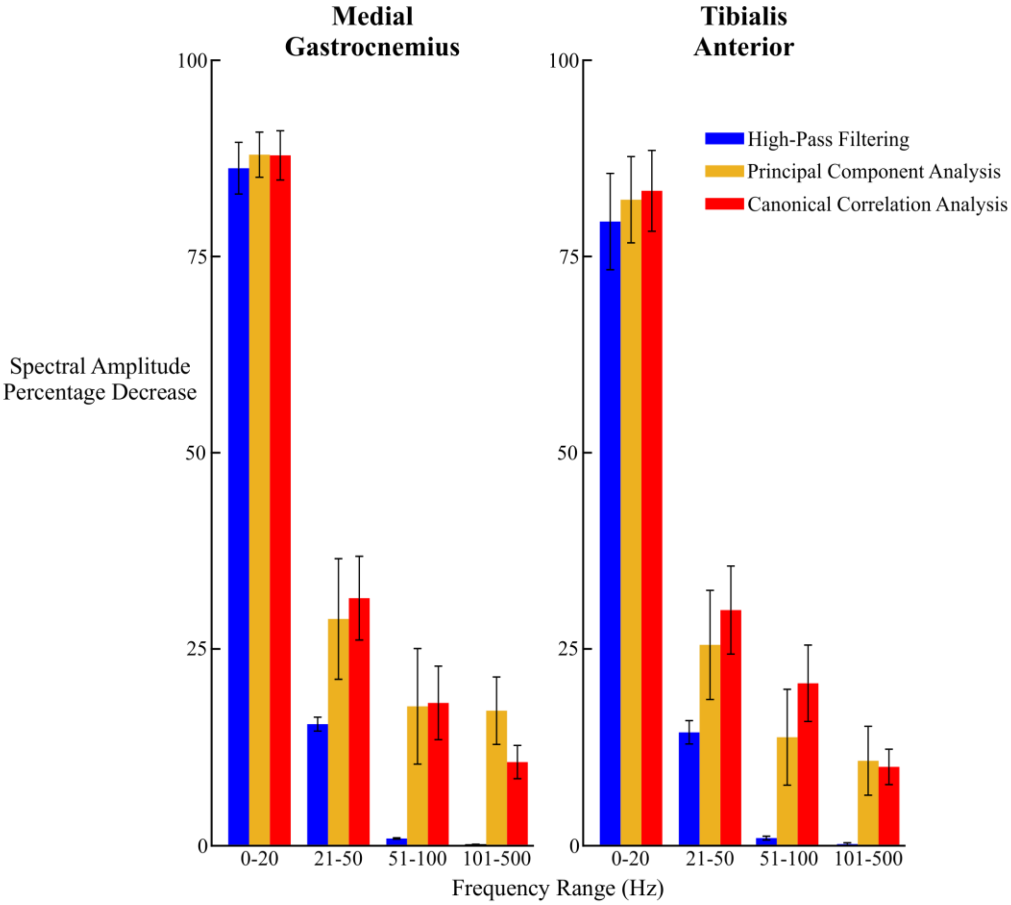

Figure 10. Average decrease in spectral amplitude between each processing method and the raw differential EMG channels in the medial gastrocnemius (left) and tibialis anterior (right) during walking at 1.6 m/s. Error bars represent standard error. Canonical correlation analysis reduced the greatest amount of signal amplitude in frequency bands associated with motion artifacts (0-20 Hz; 21-50 Hz).

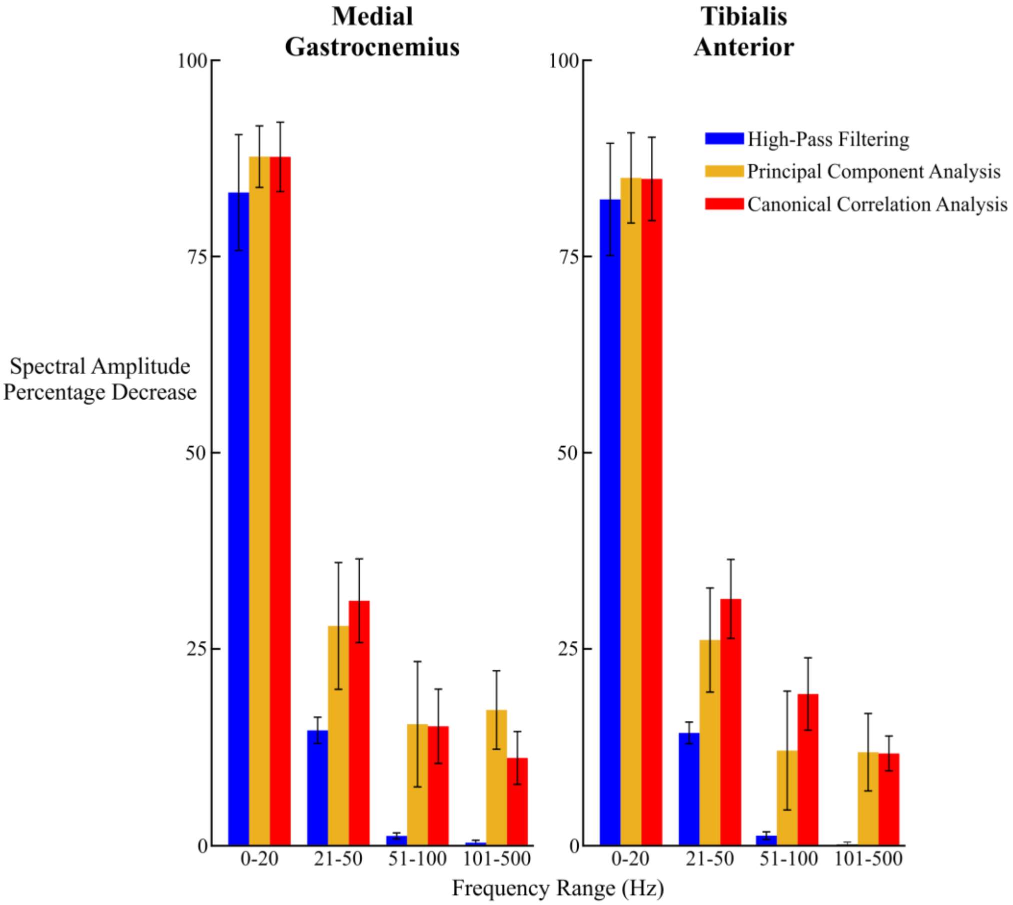

Figure 11. Average decrease in spectral amplitude between each processing method and the raw differential EMG channels in the medial gastrocnemius (left) and tibialis anterior (right) during walking at 1.2 m/s. Error bars represent standard error. Canonical correlation analysis reduced the greatest amount of signal amplitude in frequency bands associated with motion artifacts (0-20 Hz; 21-50 Hz).
